# The relevance of complement in pemphigoid diseases: A critical appraisal

**DOI:** 10.3389/fimmu.2022.973702

**Published:** 2022-08-16

**Authors:** Cristian Papara, Christian M. Karsten, Hideyuki Ujiie, Enno Schmidt, Leon F. Schmidt-Jiménez, Adrian Baican, Patricia C. Freire, Kentaro Izumi, Katja Bieber, Matthias Peipp, Admar Verschoor, Ralf J. Ludwig, Jörg Köhl, Detlef Zillikens, Christoph M. Hammers

**Affiliations:** ^1^ Department of Dermatology, University of Lübeck, Lübeck, Germany; ^2^ Department of Dermatology, Iuliu Hatieganu University of Medicine and Pharmacy, Cluj-Napoca, Romania; ^3^ Institute of Systemic Inflammation Research, University of Lübeck, Lübeck, Germany; ^4^ Department of Dermatology, Faculty of Medicine and Graduate School of Medicine, Hokkaido University, Sapporo, Japan; ^5^ Lübeck Institute of Experimental Dermatology (LIED), University of Lübeck, Lübeck, Germany; ^6^ Division of Antibody-Based Immunotherapy, Department of Medicine II, Christian-Albrechts-University of Kiel and University Medical Center Schleswig-Holstein, Kiel, Germany; ^7^ Department of Otorhinolaryngology, Klinikum rechts der Isar, Technical University Munich, Munich, Germany; ^8^ Division of Immunobiology, Cincinnati Children’s Hospital Medical Center and University of Cincinnati College of Medicine, Cincinnati, OH, United States

**Keywords:** complement, relevance, pemphigoid, bullous pemphigoid, BP, EBA, MMP, pathophysiology

## Abstract

Pemphigoid diseases are autoimmune chronic inflammatory skin diseases, which are characterized by blistering of the skin and/or mucous membranes, and circulating and tissue-bound autoantibodies. The well-established pathomechanisms comprise autoantibodies targeting various structural proteins located at the dermal-epidermal junction, leading to complement factor binding and activation. Several effector cells are thus attracted and activated, which in turn inflict characteristic tissue damage and subepidermal blistering. Moreover, the detection of linear complement deposits in the skin is a diagnostic hallmark of all pemphigoid diseases. However, recent studies showed that blistering might also occur independently of complement. This review reassesses the importance of complement in pemphigoid diseases based on current research by contrasting and contextualizing data from *in vitro*, murine and human studies.

## 1 Introduction

**Figure 1:**
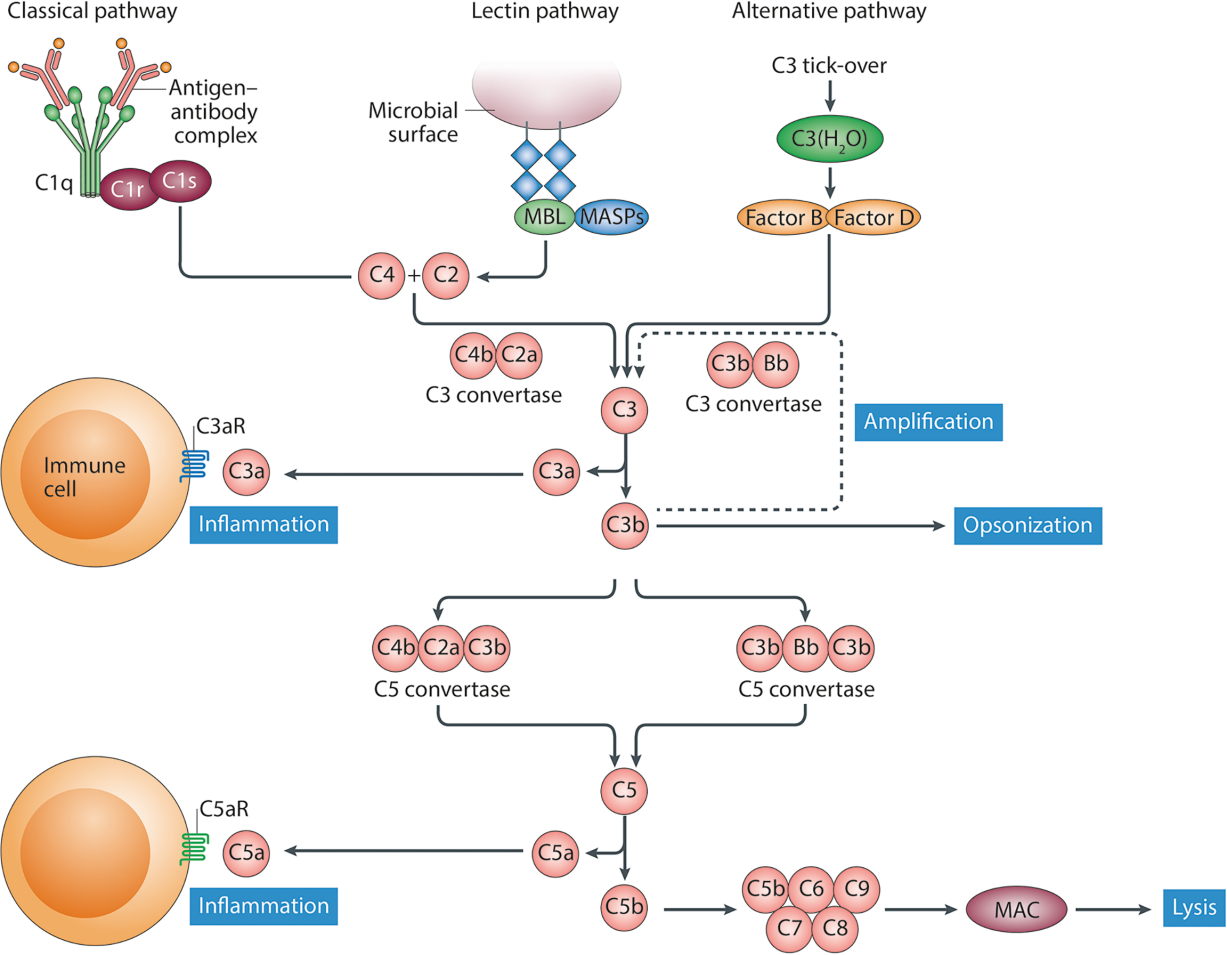
Schematic representation of the complement system. The classical pathway of complement activation is activated following to binding of the recognition molecule C1q to ligands such as immune complexes. The lectin pathway is activated following to binding of recognition molecules, such as mannose-binding lectin (MBL), collectins or ficolins, to their ligands, which include carbohydrate structures. Although the alternative pathway is initiated spontaneously, properdin (not shown) might also serve as a recognition molecule for directing activation of this pathway. Following activation *via* the initiating molecules a cascade of proteolytic activation steps leads to the formation of C3 convertases that cleave C3 into the anaphylatoxin C3a and the opsonin C3b. Next, C5 convertases generate the potent pro-inflammatory anaphylatoxins C5a and C5b, the latter of which, together with C6–C9, forms the membrane attack complex (MAC). This figure was obtained with permission from Trouw, L.A., Pickering, M.C., & Blom, A.M. Nat Rev Rheumatol. 9, 538–547 (2017), (1).

The complement system is a complex network of more than 50 proteins, which hold crucial roles in host defense against invading microorganisms as well as in tissue homeostasis, thus representing a fundamental component of the innate immune system. There are three distinct pathways of complement activation: the classical (CP), lectin (LP), and alternative pathway (AP) (**Figure 1**). Under physiological circumstances, nonself recognition in an immunoglobulin (Ig)-dependent or -independent manner triggers various proteolytic cascades that result in the activation of complement component C3. All three pathways generate a C3 convertase which elicits the immediate (C3a) and downstream (C5a) release of potent proinflammatory peptides (i. e., C5a, C3a), the opsonization of susceptible pathogens (*via* C3b and C4b), and ultimately, the lysis of microorganisms by the assembly of the membrane attack complex (MAC; composed of C5b6789) (2, 3), thereby contributing to their efficient elimination. Several soluble and membrane-bound factors regulate complement activation on different levels to protect healthy tissue from undesired damage. C1-esterase inhibitor (C1-INH), C4b-binding protein (C4BP), carboxypeptidase N (CPN1), factor H (FH), factor I (FI), protein S, and clusterin are soluble regulators of complement activation, whereas complement receptor of the immunoglobulin superfamily (CRIg), complement receptor 1 (CR1/CD35), decay-accelerating factor (DAF/CD55), membrane-cofactor protein (MCP/CD46), and protectin (CD59) mediate complement activation and regulation on the cell membrane (4).

Deficiencies in complement components or impaired complement activation pathways hinder efficient host defense responses, resulting in an increased susceptibility to infections: properdin deficiency and defects in the MAC formation are associated with an increased risk of meningococcal infections, whereas C3 deficiency is linked to recurrent infections with Streptococcus pneumoniae (5–7). In contrast, C4 deficiency is associated with systemic lupus erythematosus (SLE), but also with repeated severe herpes infections (8, 9). In children, deficiencies in mannose-binding lectin (MBL) result in recurrent bacterial respiratory infections (10). Furthermore, a defective C3-dependent opsonization increases the risk of infections with Streptococcus pneumoniae and Haemophilus influenzae, while a lack of CR3 enhances the likelihood of recurrent skin infections (2, 11).

Impaired complement system activation or regulation has been observed in many dermatological diseases, such as hereditary (12) and acquired angioedema (13), cutaneous small vessel (14, 15) and hypocomplementemic urticarial vasculitis (16), SLE (17, 18), psoriasis (19, 20), acne vulgaris (21, 22) and hidradenitis suppurativa (23). Moreover, the complement system is also involved in the pathogenesis of autoimmune blistering dermatoses (AIBD), in particular the pemphigoid group, including bullous pemphigoid (BP), epidermolysis bullosa acquisita (EBA), mucous membrane pemphigoid (MMP), pemphigoid gestationis (PG), and, to a lesser degree, the pemphigus group (24–28). C3 deposits along the dermal-epidermal junction (DEJ) are observed in approximately 90% of patients with pemphigoid diseases (PDs) (29–32). Interestingly, complement activation seems to be mainly restricted to the skin (33). In fact, the detection of C3 by direct immunofluorescence (DIF) microscopy of perilesional skin is a highly valuable diagnostic marker of all PDs. In BP, the activation of complement at the DEJ, as a result of pathogenic autoantibodies binding to collagen type XVII (BP180), initiates and maintains inflammatory processes resulting in characteristic subepidermal blistering (34). Consequently, complement components of both the CP and AP have been detected at the DEJ and blister fluid. Similarly, complement-induced separation at the DEJ has also been demonstrated in EBA, MMP and PG (35, 36). In addition, complement-fixating antibodies along the DEJ represent a characteristic feature of PG, and DIF microscopy often demonstrates rather linear C3 than IgG deposition along the basement membrane (31, 37). Regarding pemphigus, DIF microscopy shows intraepidermal deposition of IgG and/or C3, and the pathogenic IgG autoantibodies belong to the IgG1 subclass, which is a notable complement activator, but also to IgG4 (38).

By contrasting and contextualizing seemingly contradictory data from *in vitro-*, murine- and human studies we will corroborate the central role of complement, particularly in the effector phase of PDs, with a special focus on BP, EBA and MMP as prototypic PDs.

## 2 Complement-dependent pathogenic pathways in PDs

### 2.1 Experimental lines of evidence

#### 2.1.1 BP

BP is the most common autoimmune bullous disease that mainly affects the elderly, usually in the 7^th^ decade of life (24). It is caused by autoantibodies targeting two hemidesmosomal proteins, BP180 (collagen XVII, BPAG2) and BP230 (BPAG1), resulting in characteristic subepidermal blister formation (39). Clinical hallmarks range from generalized, pruritic, large skin blisters to eczematous and urticarial lesions (24).

Chorzelski and Cormane were the first to demonstrate that complement binds *in vivo* to the basement membrane of BP patients’ skin (40), followed by the substantial work of Jordon et al., which significantly contributed to our current understanding of the role of complement activation in the pathogenesis of BP. By using DIF microscopy, Jordon and co-workers showed that BP autoantibodies are able to fix not only C3 (41–43), C1q and C4 (44), but also factor B (FB) and properdin (45, 46), thus underlining the involvement of both classical and alternative complement activation pathways. Remarkably, C3 deposition was also seen in the absence of skin-bound BP autoantibodies (47), indicating a high sensitivity of the C3 staining. Furthermore, the same group demonstrated that BP blister fluids exhibited both eosinophil and neutrophil chemotactic activity, with the latter being inhibited by the use of an anti-C5 antiserum, therefore suggesting for the first time the role of complement, namely C5a, in the effector phase of BP (48). Several studies followed that corroborated these findings, to the extent that the detection of complement deposits in the skin is now considered a highly valuable and important diagnostic hallmark of BP (49–51).

Later on, Liu et al. provided experimental evidence by transferring polyclonal rabbit antibodies against murine NC14A, a homolog of the human NC16A domain of BP180, into neonatal BALB/c mice, showing that subepidermal blistering did not occur if either (i) serum complement was depleted by cobra venom factor, (ii) mice were C5-deficient, or (iii) F(ab′)2-fragments derived from the anti-murine BP180 antibody were used (52, 53). Similar results were observed in the antibody transfer model of BP in neonatal hamsters (54). These findings suggest that complement activation is indispensable for blister formation in experimental antibody transfer models of BP. Correspondingly, Nelson et al. demonstrated, in the same model, that C4-deficient mice and wild-type (WT) mice pretreated with anti-C1q-antibody were protected from disease development, thereby underscoring the importance of the classical pathway activation in the pathogenesis of PDs (55). Interestingly, the same study showed that FB-deficient mice developed delayed and less intense subepidermal blisters, suggesting a minor role of complement activation *via* the alternative pathway in experimental BP.

To reduce the shortcomings of the previously mentioned antibody transfer mouse models, both Nishie et al. and Liu et al. established humanized mouse models of BP, introducing either human BP180 (56) or replacing murine BP180NC14A with the homologous human BP180NC16A epitope cluster region (57). The CP of the complement system appeared to be pivotal also in these humanized mouse models: F(ab′)2-fragments derived from pathogenic antibodies did not induce blisters, complement C3 depletion with cobra venom factor protected from disease development, and transfer of humanized IgG1 against BP180NC16A that were previously mutated at the C1q binding site resulting in reduced C1q binding, induced less blisters compared to the unmutated humanized antigen-specific IgG1 (57, 58).

**Figure 2:**
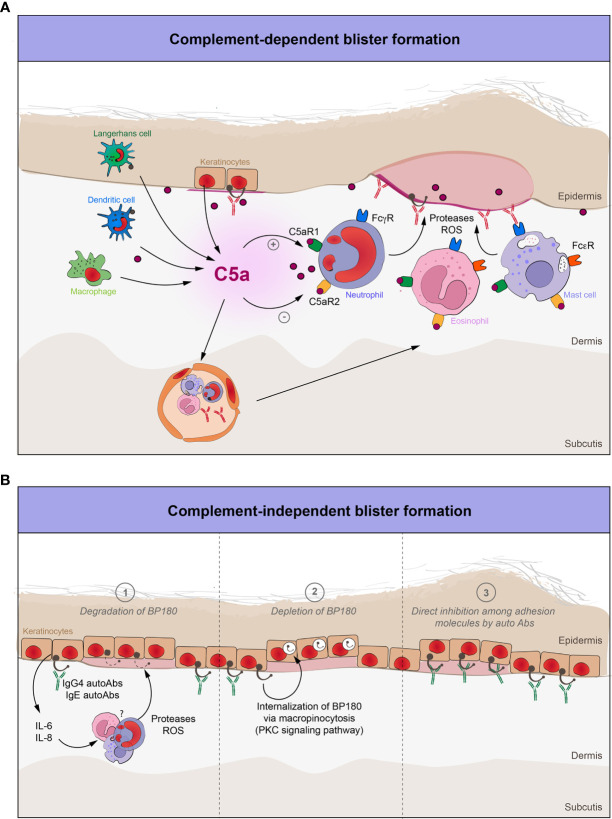
The role of complement in blister formation in pemphigoid diseases. **(A)** Complement-dependent pathways in pemphigoid diseases. Complement-fixing autoantibodies target various structural antigens of the basement membrane. The complement system is activated, thus leading to the recruitment and activation of various effector cells. C5a, which is the most potent proinflammatory peptide of the complement cascade, will interact with these cells through their C5a receptors (C5aR1, C5aR2), enabling them to release several proteases and ROS, thereby inflicting tissue damage and characteristic subepidermal blistering. **(B)** Complement-independent pathways in pemphigoid diseases. Non-complement fixing autoantibodies (IgG4) and IgE against BP180 bind to the dermal-epidermal junction and (1) degrade BP180 by increasing the expression of IL-6 and IL-8, which in turn will attract and activate neutrophils, as a major source of various proteases and ROS; (2) decrease the BP180 hemidesmosomal content by internalization of the IgG-BP180 immune complex *via* macropinocytosis; (3) directly inhibit the adhesion molecules of the dermal-epidermal junction.

Another crucial pathway underlining the importance of complement activation in BP is the interaction between C5 and its receptors, C5a receptor 1 (C5aR1) and C5a receptor 2 (C5aR2) (**Figure 2A**). By the injection of anti-BP180 IgG in adult mice, Karsten et al. recently demonstrated that C5 knockout mice had an up to 50% reduction in disease activity when compared to corresponding WT-mice (59). Unlike previous findings, this intriguing result could be explained by the use of exclusively murine models, as well as by the opposing roles of the two receptors: C5aR1-deficient mice were protected from disease development, whereas C5aR2-deficient were rather prone to develop inflammatory skin lesions (59). In addition, the pharmacological inhibition of C5aR1 by the PMX53 peptide mitigated disease development, but only when applied in a preventive setting (59).

These findings emphasize the role of complement activation in the early effector phase of the disease when neutrophils, eosinophils and mast cells are attracted by C5a to the DEJ to inflict characteristic tissue damage and subsequent blister formation (60–63). Remarkably, the depletion of neutrophils protected against BP in WT-mice, whereas the use of C5a and interleukin (IL)-8 in C5-deficient mice made them susceptible for disease development (64).

In fact, complement-dependent neutrophil infiltration in BP is contingent on prior mast cell degranulation (65), which is also induced by the activation of complement: upon the interaction between C5a and C5aR1, mast cells degranulate and release various pro-inflammatory cytokines that in turn attract neutrophils and eosinophils, as well as proteases, including the mouse mast cell protease-4 (mMCP-4) (63). The latter activates the key neutrophil protease, matrix metalloproteinase-9, both cleaving BP180 and thus, leading to blister formation (66, 67). Given this, mice deficient in mast cells, mMCP-4, or C5aR1 failed to develop BP (63, 65, 66).

In addition to neutrophils and mast cells, eosinophils appear to be involved in, at least, IgE-mediated pathology in BP, which may occur independently of neutrophils and relies on prior mast cell degranulation (61, 68). In this regard, eosinophils from most BP patients do highly express the high-affinity IgE receptor (FcϵRI) (69), and, as previously shown in a humanized FcϵRI mouse model of BP, human anti-BP180 IgE autoantibodies recruit and activate eosinophils *via* FcϵRI, resulting in eosinophil degranulation and subsequent blister formation (61). Of note, anaphylatoxins C5a and C3a can however directly induce degranulation of eosinophils *via* selective, receptor-mediated processes (70, 71), potentially connecting complement and eosinophils in human disease.

Taken together, all these data suggest that infiltration and activation of specific effector cells responsible for the characteristic BP skin pathology depend, at least indirectly, on the activated complement cascade.

#### 2.1.2 EBA

EBA is a rare autoimmune blistering disease, which is characterized by autoantibodies against type VII collagen (COL7) (72). Clinically, it can present with various phenotypes, with the classical/mechano-bullous and the non-classical/non-mechano-bullous variant being the most common forms.

Our current understanding of the role of complement in the pathogenesis of EBA is mainly relying on the use of experimental murine models which, by design, mimic human disease clinically, immunologically and histologically. EBA can be induced in mice by either transfer of human anti-COL7 IgG or rabbit anti-mouse-COL7 IgG, or by immunization of mice with an immunodominant fragment of the murine COL7 antigen (73).

Sitaru et al. showed in the active EBA model, that diseased mice presented with significantly higher C3 skin deposition compared to non-diseased animals (36). Furthermore, the complement-fixing IgG2a and IgG2b autoantibody subclasses were considerably elevated in the diseased group (36), whereas F(ab′)2-fragments of pathogenic rabbit anti-COL7 IgG did not induce blistering in the passive EBA mouse model (74). Similar results were obtained for F(ab′)2-fragments generated from affinity-purified anti-COL7 antibodies from EBA patients’ sera (75), thus underlining a key role of the IgG-fragment crystallizable region (Fc), likely mediating inflammation *via* C1q interaction and CP complement activation in these EBA models.

Experimental evidence also illustrates the involvement of the AP in passive EBA. Mihai et al. demonstrated that FB-deficient mice as well as WT-mice treated with an anti-FB antibody developed a delayed and significantly less severe blistering phenotype when compared to controls (76, 77). In contrast, C1q-, C6- and MBL-deficient mice did develop the disease, implying that the MAC formation and the LP may be dispensable for blister formation. The rather unexpected finding that C1q-deficient mice were not fully protected from disease could be explained by either the presence of non-canonical complement activation or by different Fc N-glycosylation autoantibody patterns associated with proinflammatory responses and high neutrophil activating potential and ROS release (78–80). Moreover, the interaction between T cells and antigen-presenting cells was shown to unexpectedly induce the secretion of C3 or C5, as well as the formation of their corresponding convertases, thereby generating activated complement fragments and yielding characteristic effector responses without depending on CP or AP (i.e., non-canonical complement activation) (81). Correspondingly, macrophage-generated C4 restored the altered humoral response against tumor antigens in C4-deficient mice (82). Interestingly, it was previously shown that C5a can also be generated in the absence of C3-dependent convertases (83). In addition, human keratinocytes are able to express and produce various complement proteins, including C3 (84).

Similarly to murine BP models, the C5-C5aR1 axis is engaged in downstream tissue damage and subsequent blister formation in EBA: C5-deficient mice were partially or fully protected from developing EBA in the antibody transfer model (74, 85), while the application of an anti-murine-C5 monoclonal antibody significantly reduced the blistering phenotype in this system (77). Moreover, C5aR1-deficient mice did not develop disease, and the pharmacological blockade of this receptor also led to a notable improvement of the blistering phenotype (77, 86). In contrast to the protective role of C5aR2 in experimental BP, C5aR2-deficient mice developed an attenuated disease phenotype in the antibody transfer mouse model of EBA, with C5aR2 being essential for neutrophil activation and recruitment by regulating Fcγ receptor (FcγR) expression levels in this model (59, 87). In line with these findings, Sezin et al. demonstrated that dual inhibition of both C5 and leukotriene B4 (LTB_4_) with coversin (i.e., nomacopan) suppressed disease in passive EBA much more efficiently than the inhibitor of LTB_4_ alone (88).

#### 2.1.3 MMP

MMP is a group of pemphigoid diseases with predominant mucous membrane involvement and a significant tendency towards scarring (89). Autoantibodies target various antigens, mainly BP180 and laminin 332. The mechanisms of blistering in MMP were also studied in different animal models (73). In contrast to BP and EBA, no immunization-induced mouse models have been yet described, but are in the pipeline (89). Since initial antibody transfer mouse models developed in the 90s were not completely replicating human disease, Heppe et al. recently introduced a novel model by transferring rabbit IgG against the middle and the C-terminal part of the murine laminin α3 chain, that reproduced disease clinically and immunopathologically (90–92).

Current evidence on the role of complement in experimental MMP models is scarce and contradictory at this point of time. Lazarova et al. showed that C5-deficient mice were not protected from disease development (90). Moreover, F(ab′)2-fragments from pathogenic rabbit anti-laminin 332 IgG elicited blistering in mice (91). Conversely, a recent study by Heppe et al., demonstrated that C5aR1- and FcγR-deficient mice developed little or no disease (92). The use of different mouse models for MMP could explain these discrepancies: the first study group induced MMP by passively transferring rabbit anti-laminin 332 IgG into neonatal mice, whereas the latter injected adult mice with rabbit anti-laminin 332 IgG against 2 immunodominant regions of laminin 332. Moreover, the latter mouse model was shown to fully replicate human disease, and dapsone, which is the first-choice in the therapeutic armamentarium of MMP, has proven effective in this passive MMP mouse model, thus further underlining its utility for the study of MMP (92, 93). Nevertheless, more experimental studies are needed to clarify the impact of complement in the pathogenesis of MMP.

### 2.2 Clinical lines of evidence

The detection of linear C3 deposits at the DEJ by DIF microscopy studies is routinely used to diagnose PDs and considered as the “gold standard” (31, 32, 94, 95).

In a large cohort study, Romeijn et al. demonstrated that over 80% of patients with BP showed C3c deposition along the DEJ in their (peri)lesional cutaneous biopsies (30). Moreover, this finding also significantly correlated with both clinical and serological disease activity (96). Similar results were also obtained by Ständer et al., who have shown that BP patients with C3 deposition had higher levels of seropositivity and autoantibodies (97).

Other complement components and activation factors, including C1q, C3, C3c, C3d, C4, C4d, C5, C5b-9, FB, FH, and properdin were also detected in the skin and blister fluid of BP patients, thus pointing towards the involvement of both CP and AP in the pathogenesis of human BP *in vivo* (42, 98). Interestingly, the detection of the two complement split products C3d and C4d at the DEJ by immunoperoxidase staining in formalin-fixed paraffin-embedded tissue proved as an effective novel diagnostic option for BP (99–103).

The pathogenic role of autoantibodies against BP180 is intimately intertwined with the activation of complement. Analysis of the IgG subclass distribution revealed that BP patients present a predominance of IgG1 antibodies in the skin (38). In addition, serum levels of IgG1 autoantibodies against the NC16A domain correlated with disease severity in these patients (104). Since IgG1 strongly binds complement *via* the C1q binding site of the Fc region, this adds further evidence to the decisive role of the CP in BP. Correspondingly, Sitaru et al. demonstrated that F(ab’)2 fragments of pathogenic antibodies against BP180-NC16A, which lack the Fc tail required for complement activation, did not induce dermal-epidermal separation in cryosections of human skin (105). Furthermore, Chiorean et al. recently demonstrated that the functional complement activation capacity of autoantibodies *ex vivo* in BP correlates with disease severity and autoantibody levels (96).

An IgA autoimmune response to BP180 and BP230 was also demonstrated in PDs, especially in MMP (106). It was shown to induce dermal epidermal separation *via* FcαRI-mediated neutrophil activation (107). However, there is contradictory data regarding IgA’s potential to activate complement (108, 109). With regard to PDs, neither IgA1 nor IgA2 were able to induce complement deposition at the DEJ in cryosections of human skin. However, they still could amplify the complement activation pathways *via* AP activation, which is a major source of the proinflammatory C5a (110, 111). This might explain the stronger ability of both IgA1 and IgA2 to activate neutrophils, as well as to induce blistering without the CP activation.

## 3 Complement-independent pathogenic pathways in PDs

### 3.1 Experimental lines of evidence

First evidence pointing towards direct, complement-independent blistering in PDs was presented by Kitajima et al.: Here, binding of anti-BP180 IgG to the lateral-apical cell surface of basal cells led to internalization of the BP180-IgG immune complex in both cultured keratinocytes and biopsy specimens from BP patients (112, 113). Furthermore, Iwata et al. corroborated this finding by demonstrating that pathogenic autoantibodies from BP patients not only significantly depleted the hemidesmosomal BP180 content from cultured keratinocytes, but also reduced their adhesive strength to the basement membrane, as determined in a standardized detachment assay using vibration (114). Interestingly, the α6 and β4 integrin levels of hemidesmosomes were not altered, thus underlining the putative specificity of the anti-BP180 IgG-mediated effect. The mechanism behind the internalization of the whole immune complex is attributed to the macropinocytosis pathway via calcium-dependent phosphorylation of the intracellular domain of BP180 by the protein kinase C (115, 116) (**Figure 2B**).

In line, in a neonatal BP180-humanized mouse model, Natsuga et al. were able to show that transfer of rabbit and human F(ab′)2-fragments against the immunodominant human NC16A domain of BP180 induced dermal-epidermal separation by mechanical stress, and also reduced the expression of BP180 in mouse skin as shown by immunoblotting (117). However, not all mice injected with F(ab′)2-fragments against NC16A showed skin detachment, pointing towards several synergistic pathways including Fc-mediated complement activation, potentially contributing to autoantibody-induced tissue pathology in BP (118).

Further *in vivo* data revealed that BP180 internalization and degradation *via* the ubiquitin/proteasome pathway is sufficient to induce blister formation in a C3-deficient BP180-humanized mouse model (119). Mice injected with a recombinant human IgG4 monoclonal antibody against the human NC16A domain of BP180 developed blisters, even though the IgG4 subclass does not fix complement, and also a proteasome inhibitor was added simultaneously. These results imply that BP180 internalization with subsequent hemidesmosomal weakening, followed by BP180 degradation *via* proteasome pathway may suffice for blister formation. Surprisingly, IgG4 antibodies from BP patients were found to induce dermal-epidermal separation in human cryosections, although with a much lower potential compared to IgG1 antibodies (120). Conversely, Zuo et al. suggested a protective role for IgG4 anti-NC16A antibodies in BP. He showed that the transfer of human IgG4 anti-NC16A to humanized BP180 mice inhibited human IgG1 and IgG3 induced complement activation with subsequent neutrophil infiltration, preventing both clinical and histological blistering in a dose-dependent manner (121). Correspondingly, IgG4 mitigates allergic diseases by inhibiting the activity of IgE (122). Based on these findings, it is tempting to speculate that IgG4 anti-NC16A might abrogate complement-dependent blister formation in BP. More studies are needed to fully clarify the role of IgG4 antibodies in PDs (38, 123).

Previously cited studies were nonetheless performed in neonatal mice, making data interpretation difficult at times. It was shown that neonatal mice do not entirely reproduce the clinical disease, since the majority of patients with PDs are adults and elderly, and lesions develop only with the application of friction in neonatal mice (24, 124). Moreover, there are known immunological differences between species, as well as in neutrophil function and skin physiology between neonatal and adult mice, and between murine and human skin, respectively (124, 125).

### 3.2 Clinical lines of evidence

Dainichi et al. reported two unusual BP cases with no complement deposition at the DEJ (126). Both patients had predominant IgG4 antibody involvement, which has restricted complement activating abilities. In line, approximately 20% of BP patients did not show C3 deposition at the DEJ in a large cohort of patients, and of these, the majority had prevalent IgG4 antibodies (30). Moreover, BP patients presenting with the non-blistering phenotype showed also less C3 deposition and previous studies hinted towards a IgG4 predominance in C3-negative cases (30, 127). In addition, BP-IgG4 antibodies induced subepidermal split formation *ex vivo* (120). Therefore, Dainichi et al. proposed a new entity in PDs, the so called “C3-negative BP” or “IgG4-dominant BP” (128). Given that the detection of C3 at the basement membrane is highly more sensitive than IgG for the diagnosis of BP by DIF microscopy, C3-negative DIF may require additional diagnostic methods or reconsidering the initial diagnosis (39). On the other hand, Boch et al. recently described 3 PD patients with weak or no C3 deposition, but with exclusive IgM reactivity at the cutaneous basement membrane (129). Interestingly, these patients also manifested with pruritic erythematous lesions without macroscopic or microscopic blistering. Since IgM is usually a strong inducer of complement activation, these findings further suggest that the activation of complement at the DEJ is indeed required for blister formation in PDs (108).

Both IgG1 and IgG4 against the NC16A domain of BP180 antibodies prevail in BP, and their serum levels significantly correlate with disease severity and poor prognosis (38, 104, 123). Interestingly, the IgG4 subclass was the first to be detected as well as the most prevalent in the prodromal, papular and urticarial BP variant (130). Since almost half of the IgG4 positive BP patients had also other IgG subclasses, and that IgG4 prevented IgG1 and IgG3 induced complement activation and final blister formation in mice, one might easily assume that IgG4 antibodies may play an essential role in disease induction, especially in the non-blistering phase, exerting also potential inhibitory effects as shown by Zuo et al. (121, 130). Conversely, the antibody switch to complement-fixing IgG subclasses will promote characteristic blister formation in BP (38, 107). Despite the fact that IgG4 antibodies do not fix complement, all patients with the non-blistering phenotype and IgG4 predominance still presented C3 and/or C5-9 deposition, suggesting early complement activation even with less IgG deposition at the DEJ and without blister formation (130). These findings underscore the utility of complement detection in the diagnosis of PDs, even in earlier disease stages. Recent data suggests that IgG4 might unexpectedly activate complement through both the CP and LP (131).

The report of a C4-deficient BP patient further questioned the role of complement as a prerequisite for blister development (132). Interestingly, this patient still showed linear C3 deposition at the basement membrane, implying non-canonical complement activation pathways. In contrast, C4-deficient neonatal mice were protected from BP development (55, 132). However, the administration of IL-8, which is an important polymorphonuclear neutrophil chemoattractant, restored disease susceptibility of these C4-deficient mice (55, 64). In line with this, cultured NHEKs with BP-IgG or -IgE anti-NC16A antibodies directly induced the expression of IL-6 and IL-8, whereas control IgG or BP180-deficient keratinocytes did not (133, 134). These findings suggest that the binding of BP180 IgG may induce blister formation without complement activation, rather by attracting neutrophils, which release various proteases and ROS that inflict characteristic tissue damage. Accordingly, neutrophils were shown to be indispensable for dermal-epidermal separation in both *ex vivo* murine cryosections and *in vivo* passive mouse models of PDs (64, 105, 134–136). In this sense, complement may at least maintain the inflammatory response in PD patients in a positive feedback loop pattern (39, 59, 117). On the other hand, it was shown that proinflammatory cytokines (i.e., IL-6) may actually activate complement (137, 138). More studies are needed to evaluate these complement-dependent and -independent pathways concomitantly, since one does not rule out the other, and most PD patients do feature complement fixation at the DEJ.

Notwithstanding the critical role of neutrophils in mice, eosinophil-predominant inflammatory infiltrates in the papillary dermis and eosinophilic spongiosis represent the main histological hallmarks of BP in humans (39). Furthermore, the majority of BP patients show also elevated levels of eosinophils and IgE antibodies in their sera, skin and blister fluid, respectively (139, 140). In addition, about 70% have specific IgE autoantibodies against both the NC16A and non-NC16A domains of BP180 (139, 141). Eosinophils are considered the liaison between IgE autoantibodies and skin blistering in BP. Lin et al. showed in a humanized IgE receptor mouse model of BP that IgE-mediated blistering relies on eosinophils, since eosinophil-deficient mice were protected from IgE-induced blister formation (61). Moreover, the degree of eosinophil infiltration correlated with disease severity. It seems that eosinophils are most prevalent in early urticarial lesions and that they degranulate at the basement membrane before blister formation (142). Given the fact that IgE antibodies are indicative of type I hypersensitivity responses, together with eosinophils they may be the driver of the prodromal, non-blistering phase of BP, which could not be unraveled by the clearly established IgG deposition-induced complement activation and immune effector cells recruitment paradigm. Even though IgE antibodies do not activate complement, both *in vitro* and *in vivo* data demonstrated that specific anti-BP180 IgE antibodies were still able to induce dermal-epidermal separation (62, 134, 143). However, many BP patients have both specific IgG and IgE antibodies in their sera, with the latter predominantly found on eosinophils and mast cells, and very rarely, in a discontinuous pattern along the basement membrane (144–146). Since eosinophils express the high-affinity IgE receptor FcϵRI, this might explain how anti-BP180 IgE antibodies can activate them (69). On the other hand, *in vitro* studies showed that full-length Ig as well as F(ab’)2 fragments from BP-IgG and -IgE are able to decrease the hemidesmosomes number by cytokine secretion, thereby contributing to blister formation in a FcR-independent manner (134). Moreover, BP-IgE antibodies seem to target the same NC16A domain as pathogenic IgG antibodies (146). Interestingly, Messingham et al. demonstrated by using a cryosection model of BP that eosinophil localization along the DEJ is dependent on IgG and complement deposition rather than on IgE (147). No subepidermal split was however observed in this study. To induce blister formation eosinophils required IL-5 mediated activation, also prompted by IgG deposition and complement fixation (148). Considering that most BP patients have both IgG and IgE antibodies, and that each of them may not fully explain all pathologies observed in blister formation, a potential way to integrate all the above-mentioned findings is the following: to induce blistering, specific anti-BP180 IgE autoantibodies require the presence of eosinophils, which are recruited and activated mainly as a result of IgG deposition and subsequent complement activation. Given these results, future studies should sequentially connect and integrate these distinct pathways rather than dissect and presume the existence of solely one in the intricate pathophysiology of blister formation in PDs.

## 4 Complement – useful or dispensable?

In the 90s numerous studies showed that complement is indispensable for blister formation in *ex vivo* cryosections and *in vivo* experimental mouse models of PDs (52, 53, 64, 90, 95, 149). Furthermore, the detection of C3 along the basement membrane by DIF was used since then as an important diagnostic hallmark of PDs (24, 30, 32). However, Iwata et al. questioned the necessity of complement by demonstrating that BP autoantibodies were able to deplete BP180 content in cultured keratinocytes, which in turn led to an increase in cells’ detachment implicating loss of function (114). Further research showed potential complement-independent pathways also in experimental mouse models (117, 119). However, these studies were performed in neonatal and complement knock-out mice, with the former exhibiting many limitations when compared to adult mice, and the latter being not physiologic, since patients with PDs with an additional complement component deficiency are rather an exception than the rule. Notwithstanding that Natsuga et al. demonstrated that BP antigen specific human or rabbit derived F(ab′)2-fragments were still able to induce disease, this reflects merely a theoretical potential of antibodies than a suggestion of the dispensability of complement, considering that full, complement-activating Ig molecules are prevalent in human sera and tissue, whereas F(ab’)2 fragments are artificial (117, 150).

Even though these data may refute the necessity of complement in PDs, they rather provide new insights into PD pathogenesis, suggesting that complement-dependent and -independent mechanisms may indeed coexist in patients at the same time. Even if complement is not absolutely indispensable to induce blister formation, we propose that it is essential in the amplification of characteristic inflammation and tissue damage, thus contributing to disease severity. Therefore, eliminating complement activation in PDs may significantly ameliorate disease, as data from *in vitro* and *in vivo* studies suggest (151, 152).

In view of this, different complement-targeting therapies have been specifically developed for PDs. Among these are sutimlimab and nomacopan. In a phase I trial, sutimlimab, a humanized monoclonal IgG4 antibody directed against the C1s subunit of human complement component C1, was shown to partially or completely abrogate C3 deposition along the DEJ in BP patients (153). Nomacopan (formerly known as coversin) is a bifunctional inhibitor of both C5 and leukotriene B4 (88, 154). EBA mice treated with coversin were almost completely protected from disease development mainly due to the C5-inhibitory effect of this compound (88). More recently, *Sadik et al.* demonstrated in a phase IIa clinical trial that nomacopan successfully reduced the clinical disease severity in BP patients, without any serious adverse event (152). Furthermore, several other complement-targeting treatments have been developed that so far have not been evaluated in preclinical and clinical settings (151, 154).

Piecing these data together, we conclude from the evidence published so far that complement remains an important and, in most cases, an indispensable hallmark of both human and experimental models of PDs and that complement-targeting therapies are effective and safe treatment strategies for these patients.

## Author contributions

All authors contributed to the writing and review of this manuscript. All authors approved the final version of this manuscript. All authors contributed to the article and approved the submitted version.

## Funding

This research was supported by DFG funding for the Collaborative Research Center (CRC) 1526 – *Pathomechanisms of Antibody-mediated Autoimmunity (PANTAU) – Insights from Pemphigoid Diseases *(Grant No.: 454193335), the CRU 303 P7, the Research Training Group “Autoimmune Pre-Disease” (GRK 2633), the Schleswig-Holstein Excellence-Chair Program from the State of Schleswig Holstein, the Excellence Cluster EXC 2167 *Precision Medicine in Chronic Inflammation* (TI-4), and the Sinergia *Unravel principles of self-organization in injured tissue* (CRSII5_202301/1) from the Swiss National Science Foundation.

## Conflict of interest

The authors declare that the research was conducted in the absence of any commercial or financial relationships that could be construed as a potential conflict of interest.

## Publisher’s note

All claims expressed in this article are solely those of the authors and do not necessarily represent those of their affiliated organizations, or those of the publisher, the editors and the reviewers. Any product that may be evaluated in this article, or claim that may be made by its manufacturer, is not guaranteed or endorsed by the publisher.
